# Do Women with Urinary Incontinence and Pelvic Organ Prolapse Receive Optimal First-Line Treatment?

**DOI:** 10.1007/s00192-024-05990-2

**Published:** 2024-11-11

**Authors:** Kari Bø, Marie Ellstrøm Engh, Catherine Joyce Teig, Merete Kolberg Tennfjord

**Affiliations:** 1 Department of Sports Medicine, Norwegian School of Sport Sciences, Ullevål Stadion, PO Box 4014, 0806 Oslo, Norway; 2 Department of Obstetrics and Gynecology, Akershus University Hospital, Lørenskog, Norway; 3 Faculty of Medicine, University of Oslo, Oslo, Norway; 4 The Pelvic Floor Centre, Akershus University Hospital, Lørenskog, Norway; 5 Department of Health and Training, Kristiania University College, Oslo, Norway

**Keywords:** Conservative treatment, Health care delivery, Knowledge, Pelvic floor muscle training, Physiotherapy, Surgery

## Abstract

**Introduction and Hypothesis:**

There is scant knowledge on previous pelvic floor muscle training (PFMT) in women with urinary incontinence (UI) and pelvic organ prolapse (POP) referred to hospitals. We hypothesized that women with predominately UI and POP had not received optimal primary care conservative management.

**Methods:**

This was a descriptive, cross-sectional survey among women attending a gynecological outpatient’s clinic. The questionnaire included questions about demographics, PFMT dosage, whether ability to contract had been assessed and whether the patients had used precontraction before increase in intra-abdominal pressure (“the knack”).

**Results:**

One hundred two women, mean age 52.5 (SD 13.4) years, responded; 37.3% had never been treated previously. There was no statistically significant difference in age, BMI, level of education, parity, time since last birth, SUI, or POP between the women who had been treated conservatively or not before the hospital visit. Thirty-three percent had trained with a physiotherapist and > 35% reported that their ability to contract had not been assessed or were unsure whether it had been assessed; 37% were not able to stop their urine stream; 52% reported that they performed “the knack,” with 15.7% reporting it to be effective. Reasons for not having trained the PFM before visiting the hospital included not being motivated, not knowing how to do PFMT, not being told/advised to do PFMT and not believing PFMT would help.

**Conclusion:**

The results of this study indicated that there is a need for improvement within first-line health care service for women with predominately UI and POP.

## Introduction

To date, there is level 1 evidence and recommendation A that pelvic floor muscle training (PFMT) should be first-line treatment for women with urinary incontinence (UI) [1–3]. In the general population, women with stress urinary incontinence (SUI) who perform PFMT are eight times (95% CI 4 to 19; 56% versus 6%) more likely to be cured than control groups with no or sham treatments [1]. In addition, there is now 1A evidence/recommendations for PFMT in treatment of pelvic organ prolapse (POP) symptoms grade II–III [2, 4]. No serious adverse effects have been reported during and after PFMT, and in addition to the high effectiveness and efficacy, this is the main reason why it is recommended as first-line treatment [1–5].

There is a paucity of research comparing different exercise protocols of PFMT, and which protocol that may yield the best effect, but there is evidence that both a conscious contraction of the pelvic floor muscles (PFM) before and during intra-abdominal pressure (IAP), named “the knack,” and strength training of the PFM over time is effective [5]. However, it has been shown that the effect of PFMT is significantly better if it is delivered with regular supervised training, e.g., once a week [1, 2, 6]. Supervised training, in this context, is defined as a PFMT program taught and monitored by a physiotherapist/clinician/instructor [7]. This means that the health care provider cues each PFM contraction either individually or in a group setting. Thus, if teaching and assessment is only provided at the first consultation, this is not considered supervised training [7].

Several research groups have found that more than 30% of women with PFD may not be able to perform a correct PFM contraction at their first consultation [8–11]. Moreover, within the first week postpartum, Vermantel et al. [12] found that > 50% of the new mothers were unable to contract correctly, and 45% of those who were certain they did a correct contraction were not performing it correctly. The most common errors during attempts to contract the PFM are contraction of outer pelvic muscles (e.g., the hip adductors, abdominal and gluteal muscles) [13]. In addition, Bump et al. [11] found that as many as 25% were straining/pressing down instead of squeezing and lifting inwards.

As many women have difficulties in doing correct PFM contractions, proper instruction, and assessment of ability to contract the PFM is essential. Evidence-based practice implies that surgeons ask all patients opting for surgery whether they have tried PFMT before suggesting and recommending surgery for SUI and POP [2–4]. A simple question whether PFMT has been conducted or not does not necessarily tell whether women have been taught or have performed an effective PFMT program. Knowledge of how women have been informed about PFMT, whether they have been properly assessed and whether they have received evidence-based PFMT can help improve future health service for women with UI and POP and ensure that all women are referred to professional PFM rehabilitation before consideration of surgery or other non-surgical treatment options.

The aim of this present study was to retrieve detailed information regarding the quality of former PFMT practices and other conservative treatments from women with symptoms of UI and POP scheduled for investigation and treatment at a tertiary hospital. Furthermore, to address factors why women may or may not have received PFMT or other non-surgical treatments, and the underlying reasons for not performing PFMT.

## Materials and Methods

### Design

This was a descriptive cross-sectional study conducted within a predefined 3-month period between October and December 2023 using a questionnaire to retrieve data on current and previous PFMT and other treatments in women with UI and POP.

### Participants

Consecutive women attending an outpatient gynecology clinic at a tertiary university hospital were invited to participate. The study followed the Helsinki Declaration and was approved by the Data Protection Officer at the hospital (PVO 2023_55). All participants were given oral and written information of the study and gave written consent before responding to the questionnaire.

Inclusion criteria: women aged ≥ 18 years with predominately complaint of UI and/or POP referred to the outpatient clinic and able to understand written information in the native language. Women aged < 18 years with pelvic floor pain syndromes, sexual discomfort, anal incontinence (AI), or constipation only and where the complaint was not predominately UI or POP were excluded, as were also women unable to respond to a questionnaire in the native language.

### Data Collection

All eligible women were given written information about the project after the initial investigation at the gynecology outpatient clinic. To ensure that the responses to the questionnaire would not negatively affect future investigations or treatment options the questionnaire was answered anonymously with no possibility for the researchers to link the participants to present or future medical records. Women who agreed to participate in the study and fulfilled the inclusion criteria were asked to fill in the questionnaire directly after their hospital visit and submit their responses before leaving the clinic. Experienced nurses and gynecologists provided the participants with the questionnaire. The questionnaire was given as a paper version, not electronic, and estimated time to fill in the questionnaire was 15 min.

### Questionnaire

The questionnaire contained 32 closed-ended questions covering demographic and background variables such as age, weight and height (body mass index (BMI) was estimated by the researchers), obstetric factors including parity, mode of delivery, years since last childbirth and former treatment for their condition. In addition, the women were asked in depth questions concerning previous treatment and experiences with PFMT, including frequency, intensity, and duration of training, whether they had been assessed for ability to contract, how they were assessed, whether they used “the knack,” whether using “the knack” was effective in reducing symptoms, and whether they were able to stop the urine stream.

### Statistical Analyses

SPSS version 28 was used for data analyses. Background data is reported as numbers with percentages (%) and means with standard deviation (SD). Responses to the questions are reported as numbers with %. Shapiro–Wilk test was used to test normality of distribution for continuous data. Comparison between women who had completed/not completed previous conservative treatment programs including PFMT and the knack was calculated using chi-square and *t* tests. *P* value was set to < 0.05.

## Results

Within the 3-month study period 102 women responded to the questionnaire. Background variables are detailed in Table 1. Half the women had college/university education and a large range in age, BMI, time since last childbirth, and numbers of years the patient has lived with PFD symptoms was reported. UI was the most predominant symptom followed by symptoms of POP. Other symptoms presented were AI, constipation, pelvic pain, and sexual complaints. However, in line with the inclusion criteria, these symptoms were secondary to the main complaints of UI and POP (Table 1).

**Table 1:** Background variables of the 102 women who responded to the questionnaire

	*N*	Mean and SD	Range
Age (years)	102	52.5 (13.4)	24–81
Parity (number)	102	2 (0.8)	0–4
Mode of delivery	98	Only vaginal: 85 (83.3%) CS: 3 (2.9%) Both vaginal and CS: 10 (9.8%)	
Years since last childbirth	98	21.3 (15.3)	1–59
Educational level: University/college	102	51 (50%)	
Hight, m	102	166.3 (7.0)	138–180
Bodyweight, kg	102	73.7 (13.7)	49–130
BMI (kg/m^2^)	102	26.7 (4.7)	17.3–45.2
PFD*	102		
UI POP Sexual dysfunction		88 (86.3%) 30 (29.4%) 15 (14.7%)	
AI		12 (11.8%)	
Constipation		11 (10.8%)	
Pain		11 (10.8%)	
Years lived with PFD	78	9.4 (8.1)	0.6–40

Numbers and percentages (%) and mean with standard deviation (SD) and range

*CS* cesarean section, *PFD* pelvic floor dysfunction, *POP* pelvic organ prolapse, *UI* urinary incontinence

^*^Multiple responses allowed

Ninety-four percent had heard about PFMT and 74.5% reported to have previously trained the PFM regularly. A smaller proportion (33%) had trained with a physiotherapist. Other treatment options the patients had tried included medication 29.4%, pessary 3.9%, surgery 10.8%, and electrical stimulation 19.6%. Thirty-eight (37.3%) had never been treated for their condition by health personnel before the present visit to the outpatient clinic. Concerning the question about location of the PFM, 88.2% ticked the correct response option (muscles surrounding the urethra, vagina and rectum), and 80.4% provided a correct response to the question of their understanding of a correct PFM contraction (defined as a lift & squeeze around the urethra, vagina and rectum) (Table 2). Some also ticked the incorrect response option that contracting the gluteal muscles and doing a crook lying back lift were correct PFM contractions (Table 2).

**Table 2:** Responses to questions related to pelvic floor muscle contractions

What is a correct PFM contraction*	Do not know 2.9% Contracting gluteal muscles 31.4% Contracting abdominals 7.8% Crook lying back lift 14.7% Contracting hip adductors 3.9% Lift & squeeze around urethra, vagina and rectum 80.4% Deep inspiration 2.9% Deep expiration 1.0%
Performing “the knack”	Never 12.7% Do not know 6.9% Seldom 4.9% Sometimes 14.7% Often 27.5% Every time 24.5%
Does “the knack” work	Never 7.8% Do not know 12.7% Seldom 14.7% Sometimes 27.5% Often 11.8% Every time 3.9%
Ability to stop the dribbling of urine	Yes 47.1% No 37.3% Do not know 10.8%
Whether health personnel assessed ability to contract	Yes 58 (56.9%) No 26 (25.5%) Do not know 10 (9.8%)
How ability to contract the PFM was assessed*	Vaginal palpation 56 (54.9%) Observation 14 (13.7%) Ultrasonography 15 (14.7%) MRI 0 Manometer 11 (10.8%) EMG 1 (1.0%) Do not know 11 (10’.8%)

Numbers and percentages (%)

^*^Multiple responses allowed

Fifty-two percent of the women reported that they performed “the knack” often or every time they did a movement or maneuver experienced to trigger their symptoms, but only 15.7% reported that it helped often or every time (Table 2). Thirteen of 16 (81.3%) who reported the effect of performing the knack had correctly ticked the description of a correct PFM contraction. In the women not reporting the effect of doing the knack, 82 (81.2%) ticked the correct answer. A considerable proportion reported they were not able to stop the urine stream or did not know if they were able to do this test of a correct PFM contraction. More than 35% reported that their ability to contract had not been assessed or that they did not know whether it had been assessed by health personnel (Table 2). Of those reporting to have been assessed by health personnel, the majority reported evaluation methods were vaginal palpation, ultrasonography and observation. Assessment of ability to contract the PFM was reported to have been performed by 30 of 34 (88.2%) of those who had trained with a physiotherapist.

Of the 11 (10.8%) who had previously been operated for either UI or POP, six had not been taught PFMT with a physiotherapist. Two were not able to stop the dribbling and one did not know if she was able to stop the dribble.

Table 3 presents reasons for not having done PFMT before the current visit to the hospital (*n* = 25, 24.5%). The most frequent responses were not knowing how to perform PFMT and not being motivated.

**Table 3:** Reasons for not doing PFMT*. *N* = 25

No one told me	7 (6.7%)
Cannot find a physical therapist	1 (1.0%)
Do not know how to perform PFMT	11 (10.8%)
I am not motivated	15 (14.7%)
I do not believe it can help me	7 (6.7%)

Numbers and percentages (%)

^*^Multiple responses allowed

On the question of frequency of PFMT (including both evaluation, individual and group training) by health personnel 7.8% reported one time, 12.7% 2–3 times, 10.8% 4–5 times, 4.9% 6–8 times, and 15.6% 8–12 times or more. Duration of the PFMT period varied from one week (5.9%) to > 6 months (7.8%). Contractions per set varied between 1–2 (4.9%) and > 20 (7.8%). Forty-two percent reported to have done 8–12 contractions per set. Number of sets per day varied between one set per day (44.1%) and > 3 sets per day (2.9%). Mean holding time of each PFM contraction was 8.8 s (SD 6.7).

There was no statistically significant difference in age, BMI, level of education, parity, time since last birth, and type of PDF between women who had been or not been treated conservatively before the present investigation at the tertiary hospital.

## Discussion

Although a large proportion of the women participating in the present study claimed to have trained the PFM regularly over a period of time, only one third had trained with a physiotherapist and only one third had been clinically assessed by health personnel. Many women reported to use pre-contraction before, and hold the contraction, during increase in IAP (performing “the knack”), but few reported they were able to stop the urine flow and that doing “the knack” was effective in preventing symptoms. The reported PFMT regimens varied largely, and the majority conducted regimens not following strength training guidelines for effective training [14].

Given that more than a third of women may not be able to contract the PFM correctly at their first consultation [9–11], absence of clinical assessment of ability to contract may negatively affect the success of PFMT. In the present study, a substantial number of women believed that contracting the gluteal muscles and performing a crook-lying back lift was a correct PFM contraction. Contraction of other muscle groups instead of the PFM has been found to be common in other female populations, e.g., in the postpartum period [13]. While there may be some co-contraction of the PFM during contraction of these muscles located outside the pelvis, Kruger et al. [15] found that co-contraction of the PFM during contraction of outer pelvic muscles were only minor and not recommended as a substitute for PFM contractions. The PFM is the only muscle group that is surrounding the urethra and can directly increase the urethral pressure. In addition, contraction of the PFM has also shown to make an increase in concentric needle EMG activity of the striated urethral sphincter muscle [16]. In support of this finding, MacLean et al. [17] showed that 12 weeks of supervised PFMT reduced bladder neck motion during coughing and resulted in hypertrophy of the urethral sphincter in women with SUI. The program significantly improved a 3-day bladder diary and IIQ-7 compared with the control group. Another assessor-blinded RCT in women with POP found that 6 months of supervised PFMT significantly increased PFM strength and endurance, lifted the bladder neck and rectal ampulla and narrowed the levator hiatus more in the intervention group than in the control group [18]. Although only one third of the women participating in the present study reported to have been assessed for ability to perform a correct PFM contraction, it was encouraging that most women who had trained with a physiotherapist had been assessed.

Many women participating in the present study reported they were not able to stop the urine stream, and this combined with the report that doing “the knack” did not reduce symptoms may indicate that they had not been able to perform an effective “knack” as an instant prophylactic maneuver or perform an effective PFM strength training program before they opted for further treatment. In an RCT comparing teaching of “the knack” with a control group receiving information about general exercise and nutrition, Miller et al. [19] found that performing “the knack” was significantly more effective than the control program. However, in that study, the participants had proper education and assessment of ability to perform a correct PFM contraction which seems to be a prerequisite for effective performance. In addition, a substantial number of their participants had mixed urinary incontinence and overactive bladder symptoms, and the program had a strong focus on inhibition of the desire to void and detrusor contraction making them able to reach the toilet in time. Further RCTs are necessary to rule out the effect of “the knack” in comparison with strength training of the PFM. Nevertheless, both these concepts (“the knack” and strength training of the PFMT) are based on knowledge of functional anatomy of the PFM [20, 21] and the rationale for how instant control and strength training can work [22, 23]. Both concepts have shown to be effective in treatment of SUI/MUI [1, 2] and can also be used in combination. Most women in the present study were knowledgeable of a correct PFM contraction. However, knowledge of a correct PFM contraction was equal among those reporting the knack to be effective versus those that did not report this effect. These results imply that knowledge of a PFM contraction does not seem to determine practice of PFMT or use of the knack. Furthermore, theoretical knowledge of a PFM contraction does not mean that they are able to contract correctly. Therefore, clinical assessment of ability to contract is necessary in teaching PFMT.

Notably, the training dosage varied largely between the participants, and most women had not followed general strength training recommendations of supervised training previously shown to be effective in PFMT [1, 2, 4]. The results of the present study therefore indicate that only asking the patients with symptoms of UI and POP whether they have performed PFMT or not is inadequate in order to make valid decisions for further treatment. This is especially important when the patient opts for surgery; a treatment that may have serious complications both for SUI and POP [24, 25]. More than one third of the respondents in the present study reported that they had not tried any non-surgical treatment before they were referred to a tertiary hospital, and 6 of 11 patients who already had surgery for their conditions had never had pelvic floor physiotherapy. This is not according to current international evidence-based consensus statements and recommendations in treatment algorithms [2, 3]. When asking patients whether they have previously practiced PFMT, it is essential to ask about the details of their training protocols and how they were followed-up by the health care provider. On the basis of our study’s results, we compiled a list of more detailed questions to be addressed while taking the history of patients opting for further treatment for SUI and POP (Table 4). The questions are in line with some of the items suggested in the i-Content tool for assessing therapeutic quality of exercise programs employed in RCTs [26].

**Table 4:** Suggested questions to be posed during history taking about former pelvic floor muscle training

1. Have you ever trained your pelvic floor muscles over a period of time?
2. If not, why have you never tried pelvic floor muscle training?
Follow-up question of the patients answering yes to question 1
3. When was the last time you trained your pelvic floor muscles regularly?
4. Who taught you to train your pelvic floor muscles?
5. Was your ability to contract your pelvic floor muscles assessed by a physiotherapist/health care provider?
6. How was the ability to contract your pelvic floor muscle assessed?
7. Did you train under supervision either individually or in a group?
8. How many times per week did you train you pelvic floor muscles?
9. For how long did you do the training (weeks/months)
10. Can you stop your urine stream when voiding (e.g., stop the dribbling at the end of voiding)?
11. Do you precontract and hold this contraction of your pelvic floor muscles during maneuvers (e.g. coughing, lifting, exercising) that might cause urine leakage or having a bulge or something falling out that you can see or feel in the vaginal area?
12. Does this precontraction and hold of your pelvic floor muscles stop you leaking or prevent having a bulge or something falling out that you can see or feel in the vaginal area?

The results of the present study indicate a knowledge gap concerning evidence-based recommendations for PFMT among primary health care practitioners. More education on the current recommendations for the management of these prevalent women’s health symptoms should be included in undergraduate and post-graduate curricula, especially for physiotherapists and general practitioners. A qualitative study among 28 physiotherapists found that PFMT requires comprehensive descriptions of program details for clinicians to be able to implement evidence-based practice [27].

### Strengths and Limitations

As far as we have ascertained, this is the first study asking patients with predominately UI and POP referred to a larger tertiary hospital about details of former PFMT and other treatments. More than 100 women participated in the survey. We purposely handed out the anonymous questionnaire after the consultation with the nurse or gynecologist. This was done to ensure the participants that their responses would not negatively interfere with their options for future investigation and treatment. Limitations of the study include the convenient sample and lack of knowledge of the response rate. Not knowing the response rate and the exclusion of women not understanding the native language reduces the generalizability of the results. The nurses and gynecologists were aware of this and tried their best to invite all participants that fulfilled the inclusion criteria. As we, for practical reasons, limited the study period to 3 months, we also had a restricted number of participants. The present study may therefore be considered a pilot and hopefully inspire future studies including a larger and more heterogenous population. By using an anonymous questionnaire, we could not link the patients’ report of their primary condition to the medical records and therefore rely on the patients’ report only. However, nurses and gynecologists included the patients for the study based on their referral and history taking. The questionnaire was developed by experienced experts within physiotherapy, nursing, and gynecology but was not validated. In addition, some general limitations of survey data should be acknowledged, including recall and response/social desirability bias, the latter being the wish to respond according to current known desirable practice. Response bias may be especially relevant in the present study, as the benefit of PFMT is well known through information both from health personnel and social media.

## Conclusion

The results of the present study indicate that there is a need for improvement within conservative management as first-line treatment prior to referral to a tertiary hospital for UI and POP. These results can be used to improve practices among health care practitioners, especially general medical practitioners and gynecologists referring for conservative management and among physiotherapists, providing first-line treatment for UI and POP.

## Data Availability

Data is available.

## References

[1] Dumoulin C, Cacciari LP, Hay-Smith EJC. Pelvic floor muscle training versus no treatment, or inactive control treatments, for urinary incontinence in women. Cochrane Database Syst Rev. 2018;10:CD00565. 10.1002/14651858.CD005654.pub4 PMC6516955 30288727

[2] Dumoulin C, Booth J, Cacciari L, et al. Conservative management in UI and POP in adults including neurological patients. Chapter 8. In: Cardozo L, Rovner E, Wagg A, editors., et al., Incontinence, vol. 2023. 7th ed. International Consensus on Incontinence (ICI): Bristol; 2023. p. 795–1038.

[3] NICE guideline [NG210]. Pelvic floor dysfunction: prevention and non-surgical management. 2019. https://www.nice.org.uk/guidance/ng123.

[4] Bø K, Angeles-Acedo S, Batra A, Brækken IH, Chan Y, Jorge CH, Kruger J, Yadav M, Dumoulin C. International urogynecology consultation on pelvic organ prolapse, chapter 3, committee 2; Conservative treatment of pelvic organ prolapse: Pelvic floor muscle training. Online Int Urogynecol J. 2022. 10.1007/s00192-022-05324-0. 10.1007/s00192-022-05324-0 PMC9477909 35980443

[5] Bø K. Physiotherapy management of urinary incontinence in females. J Physiother. 2020;66:147–54. 32709588 10.1016/j.jphys.2020.06.011

[6] Hay-Smith EJC, Herderschee R, Dumoulin C, Herbison GP. Comparisons of approaches to pelvic floor muscle training for urinary incontinence in women. Cochrane Database Syst Rev. 2011;12:CD009508. 10.1002/14651858.CD009508 22161451

[7] Bo K, Frawley HC, Haylen BT, Abramov Y, Almeida FG, Berghmans B, et al. An International Urogynecological Association (IUGA)/International Continence Society (ICS) joint report on the terminology for the conservative and nonpharmacological management of female pelvic floor dysfunction. Int Urogynecol J. 2017;28:191–213. 27921161 10.1007/s00192-016-3123-4

[8] Kegel AH. Stress incontinence and genital relaxation: a non-surgical method of increasing the tone of sphincters and their supporting structures. Ciba Clin Symp. 1952;4:35–51. 14905555

[9] Benvenuti F, Caputo GM, Bandinelli S, et al. Reeducative treatment of female genuine stress incontinence. Am J Phys Med. 1987;66:155–68. 3674220

[10] Bø K, Larsen S, Oseid S, et al. Knowledge about and ability to correct pelvic floor muscle exercises in women with urinary stress incontinence. Neurourol Urodyn. 1988;7:261–2.

[11] Bump R, Hurt WG, Fantl JA, Wyman JF. Assessment of Kegel exercise performance after brief verbal instruction. Am J Obstet Gynecol. 1991;165:322–32. 1872333 10.1016/0002-9378(91)90085-6

[12] Vermandel A, De Wachter S, Beyltjens T, D’Hondt D, Jacquemyn Y, Wyndaele JJ. Pelvic floor awareness and the positive effect of verbal instructions in 958 women early postdelivery. Int Urogynecol J. 2015;26:223–8. 25062656 10.1007/s00192-014-2483-x

[13] Neels H, De Wachter S, Wyndaele JJ, Van Aggelpoel T, Vermandel A. Common errors made in attempt to contract the pelvic floor muscles in women early after delivery: a prospective observational study. Eur J Obstet Gynecol Reprod Biol. 2018;220:113–7. 29202394 10.1016/j.ejogrb.2017.11.019

[14] Garber CE, Blissmer B, Deschenes MR, Franklin BA, LamonteLee MJI-Min, et al. Quantity and quality of exercise for developing and maintaining cardiorespiratory, musculoskeletal, and neuromotor fitness in apparently healthy adults: guidance for prescribing exercise. American College of Sports Medicine (ACSM) Position Stand. Med Sci Sports Exerc. 2011;43:1334–59. 21694556 10.1249/MSS.0b013e318213fefb

[15] Kruger J, Budgett D, Goodman J, Bø K. Can you train the pelvic floor muscles by contracting other related muscles? Neurourol Urodyn. 2019;38:677–83. 30592502 10.1002/nau.23890

[16] Bø K, Stien R. Needle EMG registration of striated urethral wall and pelvic floor muscle activity patterns during cough, Valsalva, abdominal, hip adductor, and gluteal muscle contractions in nulliparous healthy females. Neurourol Urodyn. 1994;13:35–41. 8156073 10.1002/nau.1930130106

[17] McLean L, Varette K, Gentilcore-Saulnier E, Harvey M-A, Baker K, Sauerbrei E. Pelvic floor muscle training in women with stress urinary incontinence causes hypertrophy of the urethral sphincters and reduces bladder neck mobility during coughing. Neurourol Urodyn. 2013;32(8):1096–102. 10.1002/nau.22343. 23861324 10.1002/nau.22343 PMC4894819

[18] Braekken IH, Majida M, Engh ME, Bø K. Morphological changes after pelvic floor muscle training measured by 3-dimensional ultrasonography: a randomized controlled trial. Obstet Gynecol. 2010;115:317–24. 20093905 10.1097/AOG.0b013e3181cbd35f

[19] Miller JM, Hawthorne KM, Park L, Tolbert M, Bies K, Garcia C, et al. Self-perceived improvement in bladder health after viewing a novel tutorial on knack use: a randomized controlled trial pilot study. J Women’s Health. 2018. 10.1089/jwh.2018.7606. 10.1089/jwh.2018.7606 PMC7583334 31800360

[20] DeLancey JO. The anatomy of the pelvic floor. Curr Opin Obstet Gynecol. 1994;6:313–6. 7742491

[21] Ashton-Miller J, DeLancey JOL. Functional anatomy of the female pelvic floor. In: Bø K, Berghmans B, Van Kampen M, Mørkved S, editors. Evidence based physical therapy for the pelvic floor. Bridging science and clinical practice. Chapter 3. Edinburg: Elsevier; 2023. p. 22–38.

[22] Bø K. Pelvic floor muscle training is effective in treatment of stress urinary incontinence, but how does it work? Int Urogynecol J Pelvic Floor Dysfunct. 2004;15:76–84. 15014933 10.1007/s00192-004-1125-0

[23] Bø K. Mechanisms for pelvic floor muscle training; morphological changes and associations between improvement in pelvic floor muscle variables and reduced symptoms of stress urinary incontinence and pelvic organ prolapse - a narrative review. Neurourol Urodyn. 2024. 10.1002/nau.25551. 10.1002/nau.25551 38979823

[24] Alvarez J, Cvach K, Dwyer P. Complications in pelvic floor surgery. Minerva Ginecol. 2013;65(1):53–67. 23412020

[25] Yeung E, Baessler K, Christmann-Schmid C, Haya N, Chen Z, Wallace SA, Mowat A, Maher C. Transvaginal mesh or grafts or native tissue repair for vaginal prolapse. Cochrane Database Syst Rev. 2024;(Issue 3):Art. No.: CD012079. 10.1002/14651858.CD012079.pub2. Accessed 14 Aug 2024. 10.1002/14651858.CD012079.pub2 PMC10936147 38477494

[26] Hoogeboom TJ, Kousemaker MC, van Meeteren NLU, Howe T, Bo K, Tugwell P, Ferreira M, de Bie RA, Van den Ende CHM, Stevens-Lapsley JE. Br J Sports Med. 2020. 10.1136/bjsports-2019-101630. 10.1136/bjsports-2019-101630 PMC8479742 33144350

[27] Slade SC, Hay-Smith J, Mastwyk S, Morris ME, Frawley H. Strategies to assist uptake of pelvic floor muscle training for people with urinary incontinence: a clinician viewpoint. Neurourol Urodyn. 2018;37:2658–68. 29797360 10.1002/nau.23716

